# Workplace telepressure and workplace cognitive failure among hospital financial personnel: a mediation analysis and latent profile analysis based on work–family conflict and self-regulatory fatigue

**DOI:** 10.3389/fpubh.2026.1898266

**Published:** 2026-07-22

**Authors:** Qian Li, Xin He, Kexin Zhang, Shu Su, Keer Ren, Qianqian Liu

**Affiliations:** 1 Department of Finance, Affiliated Hospital of North Sichuan Medical College, Nanchong, Sichuan, China; 2 School of Management, North Sichuan Medical College, Nanchong, Sichuan, China; 3 Department of Radiology, Affiliated Hospital of North Sichuan Medical College, Nanchong, Sichuan, China; 4 Department of Operations Management, Affiliated Hospital of North Sichuan Medical College, Nanchong, Sichuan, China

**Keywords:** hospital financial personnel, self-regulatory fatigue, work–family conflict, workplace cognitive failure, workplace telepressure

## Abstract

**Objective:**

Adopting both person-centered and variable-centered perspectives, this study examined the relationship between workplace telepressure and workplace cognitive failure among hospital financial personnel, along with the underlying mechanisms. Latent profile analysis was further employed to identify potential subgroups of workplace telepressure.

**Methods:**

A cross-sectional study was conducted using convenience sampling, with data collected from March to April 2026 in Sichuan Province, China. Standardized instruments were used for measurement, including the Workplace Telepressure Scale, the Work–Family Conflict Scale, the Self-Regulatory Fatigue Scale, and the Workplace Cognitive Failure Scale. From the variable-centered perspective, PROCESS Model 6 was applied to test the chain mediating effect of the chain of work–family conflict and self-regulatory fatigue, while latent profile analysis was used adopt a person-centered perspective.

**Results:**

All core variables were significantly and positively correlated. Workplace telepressure was positively associated with workplace cognitive failure among hospital financial personnel. The indirect association through work–family conflict and self-regulatory fatigue was statistically significant and was consistent with the proposed chain mediation model. The person-centered analysis identified two latent subgroups of hospital financial personnel, namely the “low-telepressure type” and the “high-telepressure type.” Financial personnel in the high-telepressure subgroup tended to exhibit higher levels of work–family conflict, self-regulatory fatigue, and workplace cognitive failure.

**Conclusion:**

By integrating dual analytical perspectives, this study addresses the limitations of prior single-perspective research and provides targeted intervention references for hospital human resource management and finance departments.

## 1 Introduction

As a critical group that bridges clinical operations and economic management, hospital financial personnel are currently undergoing a digital transformation in hospital financial management (1). On the one hand, the widespread deployment of digital infrastructures, such as hospital information systems, electronic medical records, and intelligent financial shared service centers, has gradually extended the work boundaries of hospital financial personnel from traditional offices to virtual workspaces (2, 3). On the other hand, the COVID-19 pandemic and the ensuing normalization of prevention and control measures have accelerated the adoption of remote work in the healthcare industry (4), requiring hospital financial personnel to perform core tasks through instant messaging tools, video conferencing systems, and cloud-based collaborative platforms. While modern financial work tools have substantially enhanced processing efficiency and data transparency in hospital finance (5), they have also reshaped the boundaries of work, the pace of tasks, and the psychological load of financial personnel. Consequently, the constant connectivity, immediate responsiveness, and remote accessibility brought about by digital technologies have gradually emerged as a novel source of work-related stress (6, 7). Workplace telepressure refers to the psychological tension and resource depletion that individuals experience as a result of continuous connectivity through work-related information technologies, demands for remote communication, intrusion of work into nonwork hours, and expectations of immediate feedback (8, 9). More importantly, hospital financial personnel are subjected not only to multiple occupational demands arising from frequent adjustments to medical insurance policies and compliance audit pressures (10), but also to the need to continuously respond to electronic requests from supervisory authorities, hospital administrators, and clinical departments during nonwork hours. This work pattern subjects them to telepressure that far exceeds that experienced in traditional industries. Nevertheless, few studies to date have systematically investigated telepressure among hospital financial personnel, and the underlying psychological mechanisms remain insufficiently understood.

The wellbeing and structural composition of the healthcare workforce have long been recognized as critical determinants of organizational performance, care quality, and patient safety. A substantial body of research has established that occupational strain, fatigue, and burnout among healthcare workers are closely linked to diminished performance and adverse safety outcomes. In a systematic review synthesizing 46 studies, Hall et al. (11) demonstrated that poor staff wellbeing and moderate-to-high levels of burnout were associated, in the majority of the studies reviewed, with poorer patient safety outcomes such as medical errors. At the population level, Shah et al. (12), drawing on a large national sample of registered nurses in the United States, documented a high prevalence of burnout and identified stressful work environments and excessive workloads as salient contributing factors. Beyond individual psychological states, structural workforce characteristics are likewise consequential: in a cross-sectional study across European hospitals, Aiken et al. (13) found that a richer professional nursing skill mix was associated with lower patient mortality, more favorable patient ratings, and higher quality of care. This evidence indicates that both the psychological condition and the structural configuration of the healthcare workforce carry far-reaching implications for performance and safety. Notably, however, this literature has overwhelmingly focused on frontline clinical staff—particularly nurses and physicians. In contrast, administrative and financial personnel, who increasingly operate under comparable digital connectivity and accountability pressures, have received scant empirical attention. This imbalance is consequential because errors and performance decrements among financial personnel may compromise not only fund safety and organizational efficiency but, indirectly, the resourcing and continuity of clinical care itself. Examining how a digitally driven stressor, such as workplace telepressure, shapes the cognitive functioning of hospital financial personnel extends the healthcare workforce–safety literature to a previously overlooked but managerially significant occupational group.

Workplace cognitive failure refers to the breakdown of cognitive functioning that manifests in work contexts as attentional lapses, memory omissions, action errors, biases in information judgment, and oversights in task execution (14). Prior research has demonstrated that workplace cognitive failure is closely associated with occupational safety, decision quality, and organizational effectiveness (15, 16). For hospital financial personnel, daily work places stringent demands on the timeliness and accuracy of financial activities, and any delay or error may compromise medical operations, fund safety, and organizational performance. Concurrently, remote communication tools enable financial matters to repeatedly transcend formal working hours and physical office boundaries (17), allowing work demands to intrude upon family life and personal recovery time continuously. Workplace telepressure may therefore not only undermine focused engagement with work tasks (18), but also increase the risk of workplace cognitive failure by persistently consuming cognitive resources and psychological energy (19). More critically, errors stemming from cognitive failure may not only result in financial losses but also erode physician–patient trust and the public credibility of hospitals (20). Existing research has indicated that chronic psychological stress depletes individuals’ cognitive resources and impairs executive control, thereby increasing the likelihood of cognitive failures at work (21, 22). However, research on how workplace telepressure influences cognitive failure remains relatively scarce, and the relationship between the two—as well as its underlying psychological mechanisms—has yet to be adequately explored.

Work–family conflict refers to the incompatibility between work and family roles in terms of time, pressure, or behavioral demands, which makes it difficult for individuals to simultaneously meet the expectations of both roles (23, 24). Although remote work technologies enhance work flexibility (25), in organizations characterized by an immediate-response culture and norms of constant availability, remote connectivity may paradoxically blur the boundaries between work and family (26). Financial tasks such as month-end closing, budget submission, performance accounting, and inspection audits typically involve clearly defined deadlines and unpredictable urgencies. When work-related information continuously infiltrates the family domain, individuals must not only invest additional time in handling work matters (27), but also maintain psychological attention to work concerns during family interactions (28), which may result in inadequate fulfillment of family roles, diminished support from family members, and heightened role-related guilt. Moreover, role conflict persistently consumes individuals’ emotional and cognitive resources, making it difficult to sustain stable attentional control during work (29). Specifically, unfulfilled family responsibilities can elicit guilt, irritability, and psychological preoccupation, and these affective burdens may interfere with information encoding and decision-making during work tasks. Therefore, from a theoretical perspective, workplace telepressure may be closely linked to work–family conflict because continuous work-related connectivity can increase the permeability of boundaries between work and family domains. Similarly, higher levels of work–family conflict may be associated with greater workplace cognitive failure through role-related rumination, impaired recovery, and depletion of role-management resources. Thus, this study proposes that work–family conflict may constitute a theoretically meaningful indirect association pathway linking workplace telepressure with workplace cognitive failure among hospital financial personnel.

Self-regulatory fatigue elucidates the underlying psychological mechanism linking workplace telepressure and workplace cognitive failure from the perspective of individual resource depletion. Self-regulatory fatigue refers to a state of temporary depletion of self-control resources resulting from continuous engagement in emotional regulation, attentional control, impulse inhibition, and behavioral adjustment (30, 31). In highly digitized, remotely connected work environments, hospital financial personnel must not only complete professional tasks but also continuously manage their pace of responding to online information, coordinate the demands of various departments, manage frustration arising from frequent interruptions, and maintain rigor, patience, and accuracy in high-stakes contexts. All these processes draw upon self-regulatory resources. However, prolonged and repeated self-regulation depletes psychological energy, leading to fatigue, difficulties with attentional control, and diminished error-monitoring capacity (32). Moreover, self-regulatory fatigue is closely linked with workplace cognitive failure (33). This is primarily because cognitive failure essentially reflects insufficient regulation of attention, memory, and executive control during routine work (34). As self-regulatory fatigue accumulates, individuals are more likely to rely on automatic, simplified, or experience-based processing strategies, reducing the verification and cross-checking of details, and thereby increasing the risk of accounting errors, omissions, duplicate entries, and judgment mistakes. In addition, self-regulatory fatigue diminishes task persistence and patience in problem-solving (35), making individuals more prone to cognitive avoidance and attentional drift when confronted with complex financial problems or interdepartmental disputes.

Empirical evidence from healthcare settings lends direct support to this resource-depletion pathway. Drawing on a sample of 597 nurses, Güngör and Sönmez (36) demonstrated that work intensification—conceptualized within the Job Demands–Resources framework as an escalating job demand—was significantly and positively associated with both acute and chronic occupational fatigue, and negatively associated with intershift recovery. Although occupational fatigue (the depletion of physical and mental energy) and self-regulatory fatigue (the depletion of self-control resources) are conceptually distinct, both are nested within the broader resource-depletion tradition and tend to co-evolve as cumulative work demands outpace recovery. Because workplace telepressure can be regarded as a digitally driven manifestation of intensified job demands—characterized by accelerated response expectations and the erosion of opportunities for recovery—it is reasonable to expect that, much as work intensification drives occupational fatigue, telepressure depletes the self-regulatory resources of hospital financial personnel. The downstream consequence of such depletion for work outcomes has likewise been documented: Uysal and Potas (37) reported that work fatigue was significantly associated with diminished job performance among healthcare managers, with demographic characteristics such as gender and age playing a non-trivial role in this relationship. Given that workplace cognitive failure constitutes a cognitively grounded form of performance impairment, these findings collectively reinforce the proposition that the accumulation of self-regulatory fatigue is associated with elevated workplace cognitive failure. Accordingly, workplace telepressure may be positively associated with workplace cognitive failure through higher levels of self-regulatory fatigue.

More importantly, work–family conflict and self-regulatory fatigue may jointly constitute a theoretically proposed chain indirect association between workplace telepressure and workplace cognitive failure. Telepressure provokes work–family conflict by blurring work–family boundaries, increasing work accessibility, and elevating expectations of responsiveness outside work hours. When individuals remain in a prolonged state of competing role demands, they must continuously expend self-control resources to coordinate time allocation, suppress negative emotions (38), compensate for shortcomings in family roles, and maintain work performance, which further exacerbates self-regulatory fatigue. In other words, work–family conflict serves not only as a role outcome of telepressure, but also as a critical antecedent to the depletion of self-regulatory resources. For hospital financial personnel, the need to handle online approvals, respond to departmental inquiries, or follow up on financial system issues after working hours may provoke dissatisfaction among family members and engender stress and guilt due to the inability to fulfill family responsibilities. To prevent the escalation of family conflicts while preserving professional standards at work, individuals must repeatedly engage in psychological transitions and behavioral adjustments across multiple roles. This process that continually consumes self-regulatory resources. Once self-regulatory fatigue accumulates, individuals’ capacity to sustain attention, monitor errors, integrate information, and adhere to workplace standards declines (39), ultimately resulting in elevated workplace cognitive failure. This study, therefore, proposes that the chain-mediating model of work–family conflict and self-regulatory fatigue can more precisely explicate how telepressure transforms an external work demand into role conflict and, in turn, depletes individual regulatory resources and impairs cognitive functioning.

However, this assumption may not hold under complex real-world conditions, particularly within a group such as hospital financial personnel, who are influenced by multiple contextual factors and may exhibit substantial heterogeneity in their experiences of workplace telepressure. For example, financial personnel in different positions, at different hierarchical levels, with varying years of work experience, and from different family structures may face divergent electronic information loads, response expectations, and boundary management strategies. Latent profile analysis (LPA), a representative person-centered analytic approach, identifies latent subgroups with similar characteristics within a population based on individuals’ response patterns across multiple related indicators, thereby revealing intra-group heterogeneity that is often masked by traditional variable-centered methods (40, 41). Existing research has demonstrated that employees exhibit substantial profile-based differences in stress experiences, with distinct profiles being differentially associated with mental health and job performance outcomes (42). Given that workplace telepressure encompasses multiple dimensions, including information-processing urgency and response impulsivity, this study has reasonable grounds to infer the existence of latent profile differences in this construct among hospital financial personnel.

Traditional variable-centered research paradigms typically assume homogeneity in the variable structure of the study sample and infer relationships among variables based on the basis of group-average levels (43). However, owing to differences in job responsibilities, hierarchical authority, digital proficiency, family responsibilities, organizational support, and individual boundary management capabilities, hospital financial personnel may not constitute a homogeneous population in terms of perceived workplace telepressure. Different financial personnel may exhibit latent profiles such as low-pressure type, moderate-pressure type, high-connectivity-pressure type, or globally high-pressure type. LPA identifies the heterogeneous structure of telepressure and further comparing differences across telepressure profiles in work–family conflict, self-regulatory fatigue, and workplace cognitive failure. Drawing on Conservation of Resources Theory and the self-regulatory resource model (44, 45), individuals in high-telepressure profiles are characterized by more pronounced role boundary permeation and heavier self-regulatory burdens, and are therefore expected to display higher levels of work–family conflict and self-regulatory fatigue, along with a correspondingly greater frequency of workplace cognitive failure. Conversely, individuals in low-telepressure profiles are likely to demonstrate better psychological resource conservation and superior cognitive functioning.

Based on the foregoing analysis, the present study proposes the following hypotheses:

*H1*: Workplace telepressure is significantly and positively associated with workplace cognitive failure among hospital financial personnel.

*H2*: Work–family conflict statistically mediates the association between workplace telepressure and workplace cognitive failure among hospital financial personnel.

*H3*: Self-regulatory fatigue statistically mediates the association between workplace telepressure and workplace cognitive failure among hospital financial personnel.

*H4*: Work–family conflict and self-regulatory fatigue jointly form a statistically significant chain indirect association between workplace telepressure and workplace cognitive failure.

*H5*: Hospital financial personnel exhibit heterogeneity in workplace telepressure.

*H6*: The optimal latent profiles of workplace telepressure differ significantly with respect to work–family conflict, self-regulatory fatigue, and workplace cognitive failure.

## 2 Methods

### 2.1 Study design

This study employed a cross-sectional questionnaire survey design to systematically examine the relationship between workplace telepressure and workplace cognitive failure among hospital financial personnel, along with its underlying mechanisms, and to explore latent heterogeneity in telepressure within this population. The study protocol was reviewed and approved by the Medical Ethics Committee of the Affiliated Hospital of North Sichuan Medical College (No.:2026ER320-1), and all study procedures adhered to the ethical principles set forth in the Declaration of Helsinki.

Given the cross-sectional nature of the data, the mediation model tested in this study was intended to evaluate whether the observed pattern of associations was consistent with the proposed theoretical framework, rather than to establish temporal ordering or causal effects.

### 2.2 Participants

Participants were recruited via convenience sampling from March to April 2026. To enhance the heterogeneity and representativeness of the sample, recruitment was spanned 12 hospitals distributed across Nanchong and four neighboring prefecture-level cities in Sichuan Province, China. These institutions varied systematically in tier, ownership, scale, and degree of financial informatization, encompassing tertiary, secondary, primary/community, and privately owned hospitals. The Affiliated Hospital of North Sichuan Medical College served as the host institution, while the remaining 11 hospitals participated as collaborating sites.

Specifically, the research team contacted the financial management departments of the host hospital and partner hospitals, providing detailed information regarding the study purpose, content, mode of participation, and privacy protection measures, and obtained institutional informed consent and collaborative support. With the assistance of the heads of each hospital’s finance department, electronic questionnaire links were distributed to financial personnel within their respective departments, and recruitment notices were posted in workplace messaging groups. To ensure the authenticity and quality of the collected data, IP address restrictions were imposed to prevent duplicate responses, and attention-check items were embedded within the questionnaire as quality control measures.

To ensure the representativeness of the study sample, strict inclusion and exclusion criteria were established. The inclusion criteria were as follows: (1) currently employed financial management personnel at medical institutions of various levels; (2) cumulative tenure of no less than 6 months in the current position; (3) experience handling work-related electronic information within the past 6 months (e.g., processing work information through hospital OA systems); (4) aged 18 years or older with the capacity to comprehend the questionnaire items independently; and (5) provision of voluntary written informed consent. The exclusion criteria were as follows: (1) being on long-term sick leave, maternity leave, personal leave, or full-time study leave for more than 3 months; (2) having been diagnosed with a serious psychiatric disorder within the past 3 months or currently taking medications that affect cognitive functioning; and (3) exhibiting patterned response sets or failing the attention-check items.

The minimum sample size was estimated comprehensively based on the requirements of multiple statistical analyses to ensure adequate statistical power. Following the methodological recommendations of Nylund-Gibson and Choi (46), latent profile analysis requires at least 50 participants per latent profile to obtain stable profile solutions and accurate estimates of model fit indices. Given that this study anticipated identifying three to five latent profiles, the minimum sample size required for LPA was approximately 300.

During the recruitment period, the research team contacted a total of 423 participants, meeting the minimum sample size requirement. Among them, 12 participants with less than 6 months of tenure and 11 participants exhibiting patterned responses (i.e., selecting identical responses across all items) were excluded. Consequently, the final valid sample comprised 400 participants, yielding an effective response rate of 94.56%. Of these, 191 (47.8%) were male and 209 (52.3%) were female. The average age of the participants was 36.83 ± 6.417. Detailed demographic characteristics are presented in Table 1.

**Table 1:** Differential analysis of demographic characteristics and workplace cognitive failure.

Variables	Items	Number (*N*[%])	*M*	SD	*F*/*t*	*p*	*η*^2^/Cohen *d*
Gender	Male	191 (47.8%)	3.912	0.717	2.234	0.026	0.224
	Female	209 (52.3%)	3.729	0.897			
Educational background	Technical secondary school/high school	45 (11.3%)	3.810	0.845	0.351	0.789	0.003
	Junior college	190 (47.5%)	3.824	0.809			
	Undergraduate college	109 (27.3%)	3.856	0.797			
	Master degree or above	56 (14.0%)	3.719	0.895			
Marital status	Unmarried	75 (18.8%)	3.900	0.764	0.563	0.639	0.004
	Married	275 (68.8%)	3.806	0.817			
	Get divorced	31 (7.8%)	3.682	0.972			
	Widowed spouse	19 (4.8%)	3.849	0.845			
Status of children	Have children	166 (41.5%)	3.735	0.872	−1.659	0.098	−0.168
	No children at present	234 (58.5%)	3.874	0.778			
Caring for family members^a^	Yes	251 (62.7%)	3.829	0.822	0.424	0.672	0.044
	No	149 (37.3%)	3.794	0.819			
Nature of employment	Establishment of institutions	98 (24.5%)	3.824	0.850	0.136	0.873	0.001
	Contract/appointment system	165 (41.3%)	3.792	0.783			
	Dispatch of labor services	137 (34.3%)	3.840	0.847			
Grade of hospital	Grade 3 A	140 (35.0%)	3.827	0.822	0.323	0.809	0.002
	Grade 3 B/Grade 2 A	147 (36.8%)	3.767	0.883			
	Level II and below/community health service centers	70 (17.5%)	3.868	0.714			
	Private or private hospitals	43 (10.8%)	3.865	0.769			
Finance position	A counter clerk/register clerk	126 (31.5%)	3.885	0.763	2.107	0.064	0.026
	The cashier	80 (20.0%)	3.852	0.790			
	Accounting	63 (15.8%)	3.535	0.994			
	Chief Financial officer	38 (9.5%)	3.728	0.866			
	Internal audit	73 (18.3%)	3.931	0.728			
	Other	20 (5.0%)	3.880	0.802			
Monthly income level	¥ 5,000 or less	267 (66.8%)	3.865	0.784	1.721	0.180	0.009
	5,001–10,000 ¥	72 (18.0%)	3.668	0.917			
	10,001 ¥ or more	61 (15.3%)	3.779	0.848			
Frequency of work information processing^b^	Almost never	45 (11.3%)	3.766	0.868	0.703	0.590	0.007
	Occasionally	53 (13.3%)	3.927	0.722			
	Often	114 (28.5%)	3.739	0.857			
	Almost every day	120 (30.0%)	3.813	0.826			
	All day	68 (17.0%)	3.898	0.793			

^a^Do you need long-term care for family members (e.g., disabled elderly/seriously ill patients)? ^b^How often do you handle work messages via mobile phone/wechat/Dingding etc. during non-work hours?

### 2.3 Measure tools

#### 2.3.1 Workplace Telepressure Scale

Workplace telepressure was measured using the Workplace Telepressure Scale developed by Barber and Santuzzi (8). The scale comprises six items across two dimensions—preoccupation and urge—with a representative item being, “I am concerned that I do not respond to work-related messages quickly enough.” This scale has been widely applied in Chinese populations; for instance, He et al. (47) used it to measure workplace telepressure among full-time employees and verified its sound cultural adaptability and reliability in Chinese samples. The present study employed this scale to assess workplace telepressure among financial personnel, using a 5-point Likert scale ranging from 1 (“strongly disagree”) to 5 (“strongly agree”). Total scores range from 6 to 30, with higher scores indicating greater workplace telepressure. In the present study, Cronbach’s *α* for this scale was 0.839, indicating good internal consistency. Confirmatory factor analysis was used in this study, as shown in Table 2.

**Table 2:** Confirmatory factor analysis of core study variables.

Variables	*χ*^2^/df	GFI	AGFI	RMSEA	CFI	TLI	AVE	CR
Workplace telepressure	1.880	0.986	0.968	0.047	0.991	0.985	0.550	0.859
Work–family conflict	1.817	0.937	0.921	0.045	0.974	0.971	0.543	0.957
Self-regulatory fatigue	2.629	0.912	0.888	0.064	0.948	0.941	0.516	0.950
Workplace cognitive failure	1.891	0.947	0.930	0.047	0.978	0.974	0.543	0.947

#### 2.3.2 Work–family conflict scale

Work–family conflict was measured using the Multidimensional Work–Family Conflict Scale developed by Carlson et al. (48). The scale consists of 18 items across six dimensions: time-based work interference with family, time-based family interference with work, strain-based work interference with family, strain-based family interference with work, behavior-based work interference with family, and behavior-based family interference with work. A representative item is, “The time I must devote to my job keeps me from participating equally in household responsibilities and activities.” The Chinese version of this scale was translated by Jung and Kim (49), and its cultural adaptability and reliability validated in Chinese populations. The present study used this scale to measure work–family conflict among financial personnel, employing a 5-point Likert scale (1 = “strongly disagree,” 5 = “strongly agree”). Total scores range from 18 to 80, with higher scores indicating more severe work–family conflict. In the present study, Cronbach’s *α* for this scale was 0.949, indicating excellent internal consistency. Confirmatory factor analysis was used in this study, as shown in Table 2.

#### 2.3.3 Self-regulatory fatigue scale

Self-regulatory fatigue was measured using the Self-Regulatory Fatigue Scale developed by Nes et al. (35), which assesses the depletion of self-regulatory resources at the cognitive, emotional, and behavioral levels. The scale comprises 18 items across these three dimensions, with a representative item being, “I experience recurring unpleasant thoughts.” The Chinese version was translated by Wang et al. (50) and its validity and reliability were verified in young Chinese adult populations. The present study used this scale to measure self-regulatory fatigue among financial personnel, employing a 5-point Likert scale (1 = “strongly disagree,” 5 = “strongly agree”), with reverse-coded items appropriately recoded. Total scores range from 18 to 90, with higher scores indicating more severe self-regulatory fatigue. In the present study, Cronbach’s *α* for this scale was 0.950, indicating excellent internal consistency. Confirmatory factor analysis was used in this study, as shown in Table 2.

#### 2.3.4 Workplace cognitive failure scale

Workplace cognitive failure was measured using the Workplace Cognitive Failure Scale developed by Wallace and Chen (14), which assesses unintentional lapses related to attention, memory, and action that occur in work contexts. The scale consists of 15 items across three dimensions: memory, attention, and action. A representative item is, “After leaving work, I cannot remember whether I have turned off my work equipment (e.g., computer, printer).” The present study used this scale to measure workplace cognitive failure among financial personnel, employing a 5-point Likert scale (1 = “never,” 5 = “very frequently”). Total scores range from 15 to 75, with higher scores indicating more frequent workplace cognitive failure. In the present study, Cronbach’s *α* for this scale was 0.947, indicating excellent internal consistency. Confirmatory factor analysis was used in this study, as shown in Table 2.

### 2.4 Statistical analyses

Statistical analyses and data processing were performed using SPSS 27.0 and Mplus 8.3. First, reliability analyses were conducted for all measurement scales, with Cronbach’s α > 0.7 set as the threshold. Subsequently, conventional analyses, including descriptive statistics, common-methods bias testing, univariate normality testing, and correlation analyses, were conducted. Mediation testing was conducted using PROCESS Model 6, with parameters estimated via maximum likelihood estimation and the statistical significance of indirect effects inferred using bias-corrected percentile bootstrap procedures. The number of bootstrap resamples was set to 5,000, and indirect effects were deemed statistically significant if their 95% confidence intervals did not include zero.

Latent profile analysis was conducted using the item scores of the Workplace Telepressure Scale as observed indicator variables to identify latent subgroups of hospital financial personnel exhibiting qualitatively distinct patterns of telepressure characteristics. Robust maximum likelihood estimation was employed to accommodate potential mild non-normality in the data. To prevent model estimation from converging on local maxima, each candidate model was specified with 500 sets of random start values, of which the 50 sets yielding the optimal log-likelihood values after preliminary iterations were retained for the final optimization stage. A series of models from one to five profiles were sequentially fitted, and the optimal number of profiles was determined based on a comprehensive set of criteria. Standardized Z-score plots were then used to visually present indicator score patterns across profiles, facilitating the identification of qualitative differences among them. Finally, profile-based differences in work–family conflict, self-regulatory fatigue, and workplace cognitive failure were compared.

## 3 Result

### 3.1 Common method Bias analysis

Given that the questionnaire data in this study were collected through self-report measures, common method bias may be of concern because it could artificially inflate or distort the observed associations among variables. To mitigate this risk, the present study adopted several procedural remedies during data collection, including anonymous participation, confidentiality assurances, IP restrictions to prevent duplicate responses, and embedded attention-check items to improve response quality.

Concurrently, Harman’s single-factor test was performed by entering all scale items into an unrotated exploratory factor analysis. The results indicated that five factors with eigenvalues greater than 1 were extracted in the unrotated solution, with the first factor accounting for 28.748% of the variance—well below the critical threshold of 40%. These findings suggest that no single dominant factor accounted for the majority of covariance among the study variables.

### 3.2 Descriptive statistics and correlation analysis

Descriptive statistics and correlations among the core study variables are presented in Table 3. Specifically, the skewness of the core variables ranged from −1.606 to −1.355, and the kurtosis ranged from 0.120 to 1.372, satisfying the criteria proposed by Kline (51) and approximating a normal distribution. Workplace telepressure was significantly and positively correlated with work–family conflict (*r* = 0.386, *p* < 0.01), self-regulatory fatigue (*r* = 0.281, *p* < 0.01), and workplace cognitive failure (*r* = 0.304, *p* < 0.01). Work–family conflict was significantly and positively correlated with self-regulatory fatigue (*r* = 0.365, *p* < 0.01) and workplace cognitive failure (*r* = 0.311, *p* < 0.01). Self-regulatory fatigue was significantly and positively correlated with workplace cognitive failure (*r* = 0.317, *p* < 0.01). All correlation coefficients were below 0.8, and all variance inflation factors (VIFs) were less than 5, indicating no serious multicollinearity.

**Table 3:** Descriptive statistics and correlation analysis of the core study variables.

Variables	M	SD	Skewness	Kurtosis	1	2	3	4
1. Workplace telepressure	3.709	0.855	−1.445	1.372	1			
2. Work–family conflict	3.746	0.822	−1.412	0.252	0.386^**^	1		
3. Self-regulatory fatigue	3.739	0.848	−1.355	0.120	0.281^**^	0.365^**^	1	
4. Workplace cognitive failure	3.816	0.820	−1.606	0.955	0.304^**^	0.311^**^	0.317^**^	1

^**^p < 0.01.

### 3.3 Between-group analysis

To systematically examine differences in workplace cognitive failure among hospital financial personnel with varying demographic characteristics, independent-samples *t*-tests were used for dichotomous variables, and one-way analyses of variance (ANOVAs) were used for multicategorical variables. Detailed results are presented in Table 1. The findings indicated a significant gender difference in workplace cognitive failure (*t* = 2.234, *p* = 0.026, Cohen’s *d* = 0.224). However, no significant differences were observed for educational level, marital status, parental status, family caregiving responsibilities, employment type, hospital tier, financial position, monthly income level, or frequency of work-related information processing (all *p* > 0.05).

### 3.4 Statistical chain mediation analysis of work–family conflict and self-regulatory fatigue

To further investigate the role of work–family conflict and self-regulatory fatigue in the relationship between workplace telepressure and workplace cognitive failure, the present study specified workplace telepressure as the independent variable, work–family conflict and self-regulatory fatigue as the mediating variables, and workplace cognitive failure as the dependent variable. Regression coefficients for each path were estimated using ordinary least squares, and statistical inference for indirect effects was conducted via the bias-corrected percentile bootstrap method (5,000 resamples) with 95% confidence intervals. Based on the between-group analysis results, gender was included as a control variable.

**Figure 1:**
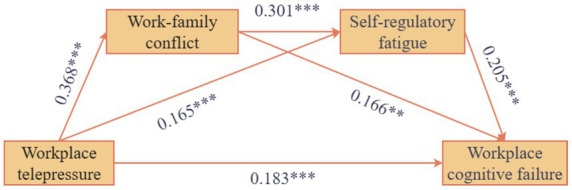
Diagram of mediation path coefficients for information search. ^**^p < 0.01;^***^p < 0.001.

The results indicated that, in Model 1 (*R*^2^ = 0.149, *F* = 69.592), workplace telepressure significantly and positively predicted work–family conflict (*β* = 0.386, 95% CI = [0.283, 0.458]). In Model 2 (*R*^2^ = 0.156, *F* = 36.796), workplace telepressure significantly and positively predicted self-regulatory fatigue (*β* = 0.165, 95% CI = [0.066, 0.261]), and work–family conflict significantly and positively predicted self-regulatory fatigue (*β* = 0.301, 95% CI = [0.209, 0.412]). In Model 3 (*R*^2^ = 0.172, *F* = 27.443), workplace telepressure significantly and positively predicted workplace cognitive failure (*β* = 0.183, 95% CI = [0.080, 0.270]), work–family conflict significantly and positively predicted workplace cognitive failure (*β* = 0.166, 95% CI = [0.064, 0.267]), and self-regulatory fatigue significantly and positively predicted workplace cognitive failure (*β* = 0.205, 95% CI = [0.104, 0.293]). Detailed results are shown in Table 4 and Figure 1.

**Table 4:** Regression analysis results.

Independent variable	Model 1				Model 2				Model 3			
	Work–family conflict				Self-regulatory fatigue				Workplace cognitive failure			
	*β*	*t*	LLCI	ULCI	*β*	*t*	LLCI	ULCI	*β*	*t*	LLCI	ULCI
Workplace telepressure	0.386	8.342^***^	0.283	0.458	0.165	3.304^***^	0.066	0.261	0.183	3.633^***^	0.080	0.270
Work–family conflict					0.301	6.029^***^	0.209	0.412	0.166	3.199^**^	0.064	0.267
Self-regulatory fatigue									0.205	4.124^***^	0.104	0.293
*R*	0.386				0.396				0.415			
*R* ^2^	0.149				0.156				0.172			
*F*	69.592^***^				36.796^***^				27.443^***^			

^**^p < 0.01; ^***^p < 0.001.

The decomposition of statistical indirect associations based on bootstrap analysis is presented in Table 5. The total association between workplace telepressure and workplace cognitive failure among hospital financial personnel was 0.292 (SE = 0.046, 95% CI = [0.202, 0.382]). Specifically, the direct statistical association between workplace telepressure and workplace cognitive failure was 0.175 (SE = 0.048, 95% CI = [0.080, 0.270]), accounting for 60.0% of the total association. Concurrently, the combined indirect association through work–family conflict and self-regulatory fatigue was 0.117 (SE = 0.031, 95% CI = [0.063, 0.185]), accounting for 40.0% of the total association. More specifically, the indirect path workplace telepressure → work–family conflict → workplace cognitive failure exhibited a statistical partial mediating effect, with an effect size of 0.061 (SE = 0.025, 95% CI = [0.018, 0.119]), accounting for 21.0%; the indirect path workplace telepressure → self-regulatory fatigue → workplace cognitive failure exhibited a statistical partial mediating effect, with an effect size of 0.033 (SE = 0.015, 95% CI = [0.009, 0.066]), accounting for 11.1%; and the indirect path workplace telepressure → work–family conflict → self-regulatory fatigue → workplace cognitive failure exhibited a statistical partial chain mediating effect, with an effect size of 0.023 (SE = 0.008, 95% CI = [0.009, 0.041]), accounting for 7.9%. These findings support H1–H4 within a cross-sectional statistical framework.

**Table 5:** Decomposition of chain mediation effects of work–family conflict and self-regulatory fatigue.

Type of effect	Effect	SE	LLCI	ULCI	Proportion of effect	Hypothesis
Total effect	0.292	0.046	0.202	0.382	100.0%	
Direct effect	0.175	0.048	0.080	0.270	60.0%	H1
Indirecet effect	0.117	0.031	0.063	0.185	40.0%	
Ind1	0.061	0.025	0.018	0.119	21.0%	H2
Ind2	0.033	0.015	0.009	0.066	11.1%	H3
Ind3	0.023	0.008	0.009	0.041	7.9%	H4

Ind 1, Workplace telepressure → work–family conflict → workplace cognitive failure; Ind 2, Workplace telepressure → self-regulatory fatigue → workplace cognitive failure; Ind 3, workplace telepressure → work–family conflict → self-regulatory fatigue → workplace cognitive failure.

Test of robustness. To further enhance the study’s accuracy, this study used age, marital status, position, and frequency of processing work-related information outside work hours as control variables, and re-applied Process Model 6 to test the chain-mediated effect of work–family conflict and self-regulatory fatigue. The results showed that work–family conflict and self-regulatory fatigue had a statistical chain mediating effect between workplace telepressure and workplace cognitive fatigue (Effect = 0.021, SE = 0.008, 95%CI = [0.008, 0.040]), which verified the above results as shown in Table 6. These results provided further support for the stability of the proposed statistical mediation pattern.

**Table 6:** Decomposition of chain mediation effects of work–family conflict and self-regulatory fatigue (Control Variables: Age, marital status, position, and frequency of processing work-related information outside of work hours).

Type of effect	Effect	SE	LLCI	ULCI	Proportion of effect	Hypothesis
Total effect	0.288	0.046	0.288	0.046	100.0%	
Direct effect	0.175	0.048	0.175	0.048	60.7%	H1
Indirecet effect	0.113	0.029	0.062	0.176	39.3%	
Ind1	0.062	0.024	0.020	0.115	21.5%	H2
Ind2	0.030	0.014	0.008	0.062	10.3%	H3
Ind3	0.021	0.008	0.008	0.040	7.4%	H4

Ind 1, Workplace telepressure → work–family conflict → workplace cognitive failure; Ind 2, Workplace telepressure → self-regulatory fatigue → workplace cognitive failure; Ind 3, workplace telepressure → work–family conflict → self-regulatory fatigue → workplace cognitive failure.

### 3.5 Latent profile analysis of workplace telepressure

To further uncover potential heterogeneity in the experience of workplace telepressure among hospital financial personnel, latent profile analysis was conducted using the scores on the six items of the Workplace Telepressure Scale as observed indicators, with models ranging from one- to five-class solutions sequentially fitted. The model fit results are presented in Table 7.

**Table 7:** Potential profile analysis indicators of remote stress in the workplace.

Profile	AIC	BIC	aBIC	Entropy	LMR (*p*)	BLRT (*p*)	Smallest proportion				
							1	2	3	4	5
1	7,494.567	7,542.464	7,504.387	–	–	–	–				
2	**6,328.11**	**6,403.948**	**6,343.66**	**0.994**	**<0.001**	**<0.001**	**18.50%**	**81.50%**			
3	6,257.613	6,361.392	6,278.892	0.990	0.003	<0.001	3.75%	14.75%	81.50%		
4	6,230.629	6,362.348	6,257.637	0.923	0.005	<0.001	3.75%	14.75%	7.75%	73.75%	
5	6,201.128	6,360.786	6,233.864	0.922	0.089	<0.001	3.75%	4.75%	10.00%	7.75%	73.75%

The bold row represents the fit index of the optimal latent model.

As shown in Table 7, the information criteria exhibited a pronounced “elbow” at the two-class solution. Relative to the one-class model, the two-class model showed a dramatic improvement in fit, with the BIC decreasing by 1,138.5 points (from 7,542.46 to 6,403.95); thereafter, the information criteria essentially plateaued, with the BIC decreasing by only 42.6 points from the two- to the three-class model and even increasing slightly from the three-class (6,361.39) to the four-class (6,362.35) model. This pattern indicates that extracting more than two classes contributed negligibly to model fit. Moreover, both the LMR and BLRT tests for the two-class model reached significance (both *p* < 0.001), and its entropy was 0.994, demonstrating exceptionally high classification accuracy.

Although the AIC and aBIC of the three- to five-class models continued to decline marginally, these solutions were rejected for three reasons. First, the smallest class in each of these solutions comprised only 3.75% of the sample (approximately 15 participants), falling well below the threshold of at least 50 participants per profile recommended by Nylund-Gibson and Choi (46), and thereby raising concerns regarding unstable estimation, weak class separation, and over-extraction. Second, the BIC—which more heavily penalizes model complexity—did not favor these more complex solutions, even increasing at the four-class model. Third, the LMR test for the five-class model was non-significant (*p* = 0.089), indicating that the five-class solution did not significantly improve fit relative to the four-class model, while the additional micro-classes lacked distinct and interpretable response patterns.

Notably, although the two-class solution yielded an unbalanced class distribution (18.5% vs. 81.5%), this imbalance does not undermine the validity of the solution: the smallest class (*n* = 74) substantially exceeded the 50-per-class stability threshold, and the near-perfect entropy (0.994) confirmed that the two profiles were cleanly and reliably separated. Unequal profile sizes are common and statistically acceptable in latent profile analysis, as class proportions are not required to be balanced. Accordingly, the two-class model was selected as the optimal latent profile solution for workplace telepressure among hospital financial personnel, supporting H5.

**Figure 2:**
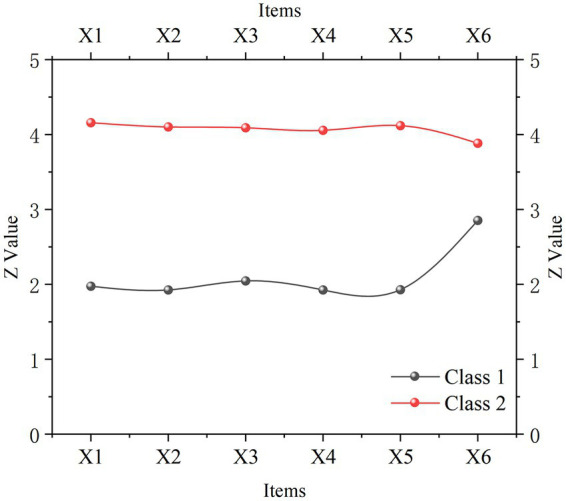
Profile of remote stress in the workplace. Class 1, Low Telepressure; Class 2, High Telepressure.

Based on the classification results of the two-class model, profile plots of workplace telepressure were generated using Origin 2021, as illustrated in Figure 2. Class 1 comprised 74 participants, accounting for 18.50% of the total sample, and was labeled the “Low Telepressure” group. This group’s scores on all telepressure items were generally below the sample mean, suggesting that these individuals were less likely to exhibit persistent concern about the speed of replying to work messages, urges for immediate responses, or psychological tension arising from connectivity to work-related information. Class 2 comprised 326 participants, accounting for 81.50% of the total sample, and was labeled the “High Telepressure” group. This group’s scores on all telepressure items were generally elevated, indicating that these individuals were more likely to perceive sustained connectivity pressure from work-related information technology, displayed stronger psychological preoccupation and behavioral urges regarding response speed to work messages, and may have maintained higher levels of work accessibility outside working hours.

### 3.6 Between-profile comparisons of the optimal latent profile solution

The present study further compared the differences between the workplace telepressure profiles in work–family conflict, self-regulatory fatigue, and workplace cognitive failure to examine whether distinct telepressure experience patterns corresponded to differing levels of role conflict, psychological resource depletion, and cognitive functioning failure. Independent-samples *t*-tests were employed to compare the mean differences between the two classes on the core outcome variables, as presented in Table 8.

**Table 8:** Differential analysis of the optimal latent profile.

Variables	Index	Class 1	Class 2
Work–family conflict	*M*	3.191	3.872
	SD	1.026	0.712
	*t*	−6.788	
	*p*	<0.001	
	Cohen *d*	−0.874	
Self-regulatory fatigue	*M*	3.165	3.869
	SD	0.999	0.752
	*t*	−6.802	
	*p*	<0.001	
	Cohen *d*	−0.876	
Workplace cognitive failure	*M*	3.304	3.933
	SD	1.036	0.715
	*t*	−6.233	
	*p*	<0.001	
	Cohen *d*	−0.801	

Class 1, Low Telepressure; Class 2, High Telepressure.

The between-profile comparisons revealed that individuals in the High Telepressure group scored significantly higher than those in the Low Telepressure group on work–family conflict (*t* = −6.788, *p* < 0.001), self-regulatory fatigue (*t* = −6.802, *p* < 0.001), and workplace cognitive failure (*t* = −6.233, *p* < 0.001), with all three differences yielding substantial effect sizes. Thus, the High Telepressure profile corresponded to more severe role boundary conflict, more pronounced depletion of self-regulatory resources, and more frequent cognitive functioning failures, supporting H6.

## 4 Discussion

### 4.1 Variable-centered findings: indirect associations linking Telepressure to cognitive failure

Workplace telepressure among hospital financial personnel was significantly and positively associated with predict workplace cognitive failure. This finding aligns with the broader occupational-stress literature showing that sustained job stressors deplete limited cognitive resources and undermine attentional control, working memory, and executive monitoring (22, 52). It also extends this literature to a digitalized, high-accountability context: in financial work, where accuracy, timeliness, and standardization are non-negotiable, remote-work technologies improve information flow but simultaneously intensify persistent accessibility and immediate-response expectations (53), preventing full disengagement from the work role and leaving the cognitive system under chronic load.

Beyond this overall association, two complementary mechanisms clarified how telepressure translates into cognitive failure. First, work–family conflict mediated the relationship, indicating that telepressure operates partly by eroding work–family boundaries rather than acting solely as a direct cognitive load. When work demands repeatedly intrude into non-working hours, they compress recovery time and elicit guilt, irritability, and rumination that spill back into task processing. This is consistent with boundary-theory accounts of role-boundary permeability (54), and complements prior telepressure–recovery studies by showing that the cost of constant connectivity is not confined to the home domain but feeds back into work-domain cognition. Second, self-regulatory fatigue mediated the relationship, supporting a resource-depletion account: continuously monitoring messages, prioritizing information (55), and suppressing interruption-related negative affect requires sustained self-control, the depletion of which manifests as lapses in attention, weakened error monitoring, and impaired impulse control.

More importantly, work–family conflict and self-regulatory fatigue formed a statistically significant chain indirect association. This pattern suggests that work–family conflict may not only co-occur with telepressure but may also be linked to self-regulatory fatigue, which is in turn associated with workplace cognitive failure. Reconciling competing work and family role demands requires continuous self-control, emotional regulation, and attentional switching. Such demands may be associated with reduced regulatory resources, and lower regulatory resources may correspond to weaker attentional control, reduced standard adherence, and diminished error detection during financial tasks. This chain offers a more integrated process explanation than the parallel-mediation models that dominate existing work, which typically treat boundary conflict and resource loss as independent outcomes.

The decomposition results refine this interpretation in two ways. The total indirect association accounted for 40.0% of the total association between workplace telepressure and workplace cognitive failure, indicating that boundary-related conflict and self-regulatory fatigue are important correlates in this relationship. The persistent direct association suggests that other unmeasured factors may also be involved, such as task fragmentation from frequent interruptions, information overload from excessive online communication, and judgment burden under incomplete or delayed remote information. Future longitudinal or experience-sampling research is needed to determine whether these factors operate as temporal mechanisms linking telepressure to subsequent cognitive failure.

A long line of research on work characteristics and scheduling has shown that the temporal architecture of work, how, when, and for how long employees work, and how much recovery time intervenes, is a modifiable occupational demand that shapes wellbeing and performance. In their review of shift-work characteristics, Dall’Ora et al. (56) reported that features such as long shifts, night work, and overtime are associated with heightened fatigue, burnout, and impaired performance, largely through the erosion of adequate recovery. Workplace telepressure can be productively understood as a contemporary, digitalized counterpart to these traditional scheduling features: whereas shift characteristics structure the physical boundaries of work time, telepressure dissolves them, extending psychological work availability into ostensibly non-working hours and compressing the recovery periods that buffer cognitive resources. Crucially, both classes of demand appear to operate through a shared mechanistic core—insufficient recovery and the cumulative depletion of regulatory resources—which converges closely with the self-regulatory fatigue pathway identified in the present study. Conceptualizing telepressure not as an isolated novel construct but as an emergent member of the work-characteristics family thus integrates our findings with an established body of evidence and underscores that, like scheduling, telepressure is in principle amenable to organizational design and modification.

It is worth noting that the between-group analysis revealed a significant gender difference in workplace cognitive failure, while no significant differences emerged across other demographic variables. This suggests that, within the population of hospital financial personnel, the occurrence of cognitive failure may not be primarily determined by traditional demographic characteristics, but rather more closely linked to specific work stress experiences, role boundary states, and psychological resource levels. Therefore, compared with risk assessments based solely on external characteristics such as job position, education, or income, attending to employees’ subjective stress experiences and changes in psychological resources within remote work contexts may be more conducive to identifying the risk of cognitive failure.

### 4.2 Person-centered findings: a normalized, heterogeneous risk structure

The High Telepressure profile accounted for 81.50% of participants, whereas the Low Telepressure profile accounted for 18.50%. As hospital information systems and online approval workflows extend financial work from fixed offices into device- and cloud-based virtual spaces, telepressure appears to have become a normalized stressor for the majority, not an episodic experience confined to a vulnerable few.

From a practical standpoint, the small but clearly delineated Low Telepressure profile (18.5%) constitutes a valuable reference subgroup. Rather than representing residual noise, these individuals, who maintained lower connectivity pressure despite operating within the same digitalized environment, may possess more effective boundary-management strategies, stronger psychological detachment, or more supportive role conditions. Identifying and learning from this protective profile can inform the design of organization-wide interventions, while the dominance of the High Telepressure profile underscores the need for population-level, rather than merely individual-level, mitigation measures. In this sense, the unbalanced two-profile structure offers more actionable managerial insight than a more evenly split but less stable multi-class solution would have provided.

Further between-group comparisons revealed that individuals in the High Telepressure profile scored significantly higher than those in the Low Telepressure profile on work–family conflict, self-regulatory fatigue, and workplace cognitive failure, with all effect sizes reaching substantial magnitudes. This finding further indicates that distinct telepressure profiles correspond to varying levels of psychological and work-related cognitive risk. Specifically, individuals in the High Telepressure profile are more susceptible to the influence of continuous online presence and rapid response norms, with their work tasks more likely to extend beyond formal working hours into the family domain, thereby leading to higher levels of work–family conflict. Moreover, these individuals are required to engage more frequently in attentional control, emotion regulation, and behavioral inhibition, rendering them more prone to self-regulatory fatigue. When role conflict and resource depletion co-occur, individuals’ working memory, error monitoring, and executive control capacities further decline, ultimately manifesting as elevated levels of workplace cognitive failure.

The person-centered findings further extend the explanatory framework of workplace telepressure research. Existing studies have predominantly examined the antecedents and consequences of telepressure from a variable-centered perspective, emphasizing the overall associations between telepressure and employee recovery, sleep, job burnout, emotional exhaustion, or job performance (8, 57). However, the present study demonstrates that telepressure does not manifest in a uniform manner across all individuals, but rather can present as latent subgroups characterized by differentiated risk levels. This individual-level variability in telepressure may be particularly pronounced among hospital financial personnel, an occupational group that simultaneously bears responsibilities for administrative service, financial management, compliance control, and data processing.

### 4.3 Practical implications

The findings of the present study offer important practical implications for hospital administrators, human resource departments, and financial management departments. First, hospitals should incorporate workplace telepressure into their occupational health risk management systems for employees. Administrators should rigorously evaluate its potential effects on employees’ work boundaries, psychological recovery, and cognitive performance. Therefore, hospitals should regularly assess the telepressure levels of financial personnel and establish psychological risk monitoring mechanisms tied to digital workload, in order to identify employees in the High Telepressure profile in a timely manner.

A key source of telepressure is employees’ persistent concern and impulsivity regarding rapid replies to work messages. To address this, hospitals can institutionally specify the priority levels, response timeframes, and after-hours handling rules for various types of work-related information. Concurrently, administrators should refrain from issuing non-urgent tasks during non-working hours and avoid treating “always-on” availability as an implicit performance expectation. Establishing more reasonable digital communication boundaries can therefore reduce employees’ sustained vigilance toward work messages and lessen the encroachment of telepressure upon family life and psychological recovery.

In addition, hospital financial departments should optimize work processes and task allocation to mitigate the risk of concentrated telepressure during peak periods. Administrators can reduce the frequency of *ad hoc* online communication and repeated follow-up requests by planning task milestones in advance, clarifying division of responsibilities, establishing backup positions, optimizing approval workflows, and refining information system functionalities.

Fourth, the recovery of employees’ self-regulatory resources and psychological support should be reinforced. The present study found that self-regulatory fatigue is an important psychological mechanism linking telepressure with cognitive failure. Hospitals can enhance financial personnel’s capacity for psychological resource recovery through occupational mental health training, stress management courses, mindfulness training, recovery experience interventions, and time management training. Furthermore, administrators should attend to early warning signs such as persistent fatigue, declining attention, emotional irritability, and increased work errors, and provide supportive communication and work adjustments in a timely manner. For employees who remain in the High Telepressure profile over the long term, individualized psychological counseling or employee assistance services may be considered to prevent resource depletion from progressing into job burnout or more severe mental health problems.

The two-profile structure provides hospital administrators with a concrete, screening-based framework for allocating intervention resources rather than applying uniform measures across all financial personnel. As a first step, the six-item Workplace Telepressure Scale can be embedded into routine occupational-health surveillance, and the same item-response patterns that distinguished the two profiles in this study—generally below- versus above-average endorsement across all items—can be used as a practical rule of thumb to triage employees into the Low- and High-Telepressure groups. At the boundary level, administrators can prioritize measures that target the proximal driver—blurred work–family boundaries—such as tiered message-priority protocols, explicit after-hours response norms, and “communication curfews” for non-urgent matters. At the resource level, because this profile was also characterized by pronounced self-regulatory fatigue, boundary measures can be paired with recovery-oriented support (e.g., protected detachment periods, recovery-experience and mindfulness training, and workload re-balancing during month-end closing and audit peaks). At the individual level, employees who remain in this profile despite organizational measures may warrant more intensive, personalized support such as employee-assistance counseling, so as to interrupt the progression from resource depletion toward cognitive failure and burnout.

### 4.4 Limitations and future research directions

Despite the aforementioned findings, the present study has several limitations. First, the study employed a cross-sectional survey design, which precludes conclusions regarding temporal ordering and causality. Although the statistical mediation model was theoretically grounded and the observed data were consistent with the proposed framework, the results cannot establish that workplace telepressure causes work–family conflict, self-regulatory fatigue, or workplace cognitive failure. Reverse or reciprocal relationships are also plausible. For example, employees with more frequent cognitive failures may experience reduced work efficiency, require more time to complete tasks, and consequently continue handling work-related messages during non-working hours, thereby reporting higher workplace telepressure. Similarly, individuals with higher self-regulatory fatigue or greater work–family conflict may perceive digital communication demands as more stressful. Future research should employ longitudinal designs, multi-wave panel studies, diary methods, experience sampling, or cross-lagged models to examine temporal sequencing and causal direction among telepressure, role conflict, self-regulatory fatigue, and cognitive failure.

Second, the data in the present study were primarily derived from self-report questionnaires, which may be subject to common method bias, social desirability bias, recall bias, transient emotional states, and individual response tendencies. Although self-report measures are appropriate for assessing subjective psychological experiences such as workplace telepressure, work–family conflict, and self-regulatory fatigue, they may inflate or distort associations among variables when all constructs are measured from the same source at the same time. In this study, we adopted several procedural remedies to reduce this risk, including anonymous responding, confidentiality assurances, IP restrictions, and attention-check items. We also used Harman’s single-factor test as a preliminary statistical diagnostic because it is a widely used and easily interpretable screening method in cross-sectional questionnaire research, especially when no theoretically appropriate marker variable was included in the original survey design. The result showed that the first unrotated factor accounted for 28.748% of the variance, suggesting that a single dominant method factor was unlikely to account for the majority of covariance among variables. Nevertheless, Harman’s single-factor test has well-recognized limitations, including limited sensitivity and an inability to detect more complex or construct-specific sources of common method variance. Therefore, the possibility of residual common method bias cannot be completely ruled out. Future studies should incorporate more rigorous approaches, such as marker-variable techniques, confirmatory factor analysis with an unmeasured latent method factor, temporal separation of measurements, multi-source data, supervisor or peer ratings, objective error records, system log data, and behavioral indicators of message response patterns.

These findings suggest that no single dominant factor accounted for the majority of covariance among the study variables. The use of convenience sampling limits generalizability. Although the sample included 400 financial personnel from 12 hospitals differing in tier, ownership, scale, and informatization level, participants were recruited non-randomly and were geographically concentrated in Sichuan Province. The findings may therefore not fully generalize to hospital financial personnel in other regions or healthcare systems. Future research should adopt multi-region, multi-center, stratified random sampling and use multilevel modeling to account for the nesting of employees within hospitals.

The present study primarily examined the mediating roles of work–family conflict and self-regulatory fatigue; however, the mechanisms through which workplace telepressure influences cognitive failure may be more complex. Telepressure may further influence cognitive functioning through pathways such as information overload, technostress, declines in sleep quality, and insufficient psychological detachment. Moreover, individuals may differ in their resource conservation capacities and stress coping styles, with factors such as psychological capital, organizational support, recovery experiences, and emotion regulation strategies potentially moderating the impact of telepressure. Future research could further construct moderated mediation models or more complex integrative models to elucidate the boundary conditions and multiple mechanisms underlying the influence of telepressure on workplace cognitive failure.

The present study did not finely differentiate the specific work contexts of various positions within hospital financial departments. This was primarily due to the considerable heterogeneity of positions within hospital financial departments, with personnel in different roles potentially confronting distinct information loads, risk responsibilities, and remote communication counterparts. Although the between-group analysis in the present study found no significant differences in workplace cognitive failure across various financial positions, this does not imply that the positional context is unimportant. Future research could employ qualitative interviews, mixed-methods research, or multilevel linear models to further analyze how job responsibilities, organizational hierarchy, departmental support, and work process characteristics influence telepressure and its consequences.

## 5 Conclusion

By integrating variable-centered and person-centered analytical perspectives, this study found that workplace telepressure was positively associated with workplace cognitive failure among hospital financial personnel. Work–family conflict and self-regulatory fatigue showed significant statistical indirect associations in this relationship, and the chain mediation pattern was consistent with the proposed theoretical framework. Latent profile analysis identified two workplace telepressure profiles: a low-telepressure profile and a high-telepressure profile. Compared with the low-telepressure profile, the high-telepressure profile reported higher levels of work–family conflict, self-regulatory fatigue, and workplace cognitive failure. These findings suggest that telepressure may represent an important digital-work-related psychological risk factor among hospital financial personnel. Nevertheless, because the study used a cross-sectional design, the results should be interpreted as associative rather than causal. Future longitudinal and multi-source research is needed to verify the temporal and causal nature of these relationships.

## Data Availability

The raw data supporting the conclusions of this article will be made available by the authors, without undue reservation.
